# Severe Octreotide-Induced Immune Thrombocytopenia With Bone Marrow Findings Suggestive of Acquired Amegakaryocytic Thrombocytopenic Purpura in a Cirrhotic Woman: An Unusual Case Report

**DOI:** 10.7759/cureus.96967

**Published:** 2025-11-16

**Authors:** Erick Crespo Solís, Alvaro Graniel Flores, Juan C Pavón Ortega, Lucía J Osorno Solís, Iaraah Montalvo Gordon, Alejandra Álvarez Salinas, Lauro F Amador Medina

**Affiliations:** 1 Hematology, Christus Muguerza Hospital Faro del Mayab, Mérida, MEX; 2 Anesthesiology, Universidad de Guanajuato, León, MEX; 3 Hematology, Hospital Regional de Alta Especialidad del Bajío, Servicios de Salud del Instituto Mexicano del Seguro Social para el Bienestar (IMSS Bienestar), León, MEX; 4 Research, Unidad Médica de Alta Especialidad (UMAE) Hospital de Especialidades No. 1, Centro Médico Nacional (CMN) Bajío, León, MEX

**Keywords:** acquired amegakaryocytic thrombocytopenic purpura, chronic thrombocytopenia, drug-induced immune thrombocytopenia, metabolic liver cirrhosis, octreotide-associated thrombocytopenia

## Abstract

Drug-induced immune thrombocytopenia (DIT) is a rare but potentially life-threatening condition that is often underrecognized in clinical practice. Octreotide, a somatostatin analogue used to control GI bleeding, has rarely been associated with DIT. We describe an 80-year-old woman with metabolic cirrhosis and chronic thrombocytopenia who developed profound thrombocytopenia and severe bleeding shortly after initiating octreotide. Bone marrow evaluation revealed marked megakaryocytic hypoplasia, initially suggestive of acquired amegakaryocytic thrombocytopenic purpura (AATP). Antiplatelet antibodies were detected, and the patient responded rapidly to dexamethasone, intravenous immunoglobulin, and eltrombopag. Although the presentation resembled classic DIT, the marrow findings and clinical course suggested overlapping features of AATP and bone marrow aplasia. This case underscores the diagnostic challenges of thrombocytopenia in cirrhosis, the potential suppressive effect of octreotide on megakaryopoiesis, and the importance of early marrow assessment and immunosuppressive therapy in severe cases.

## Introduction

Drug-induced immune thrombocytopenia (DIT) is an uncommon but clinically important condition that is often underrecognized. First described in patients treated with quinine for malaria [1], DIT may result from exposure to various medications, although not all cases involve immune-mediated platelet destruction [2,3]. Its estimated incidence is about 10 cases per million population, with higher prevalence among hospitalised elderly patients and users of certain antibiotics [2]. Clinically, DIT typically develops one to two weeks after exposure but may occur sooner in patients with preexisting antibodies. Severe cases can present with wet purpura, GI or CNS bleeding, and, less commonly, fever or nausea. Diagnosis remains largely clinical due to limited availability and uncertain reliability of serologic testing. Recognition of the temporal relationship between drug exposure and symptom onset is crucial, and tools such as the Aster criteria and the Naranjo algorithm can help establish a drug-induced aetiology [1,4].

Octreotide, a somatostatin analogue used for GI bleeding, has rarely been associated with DIT. Reported cases are few and generally describe a reversible course after discontinuation [5,6]. We report a case of severe thrombocytopenia following octreotide administration in a cirrhotic patient whose bone marrow findings initially suggested acquired amegakaryocytic thrombocytopenic purpura (AATP). However, the clinical course and treatment response were more consistent with superimposed bone marrow hypoplasia. This case underscores the diagnostic complexity of thrombocytopenia in cirrhosis and the importance of considering rare immune-mediated drug reactions, as well as performing bone marrow evaluation to assess megakaryopoiesis.

## Case presentation

**Figure 1:**
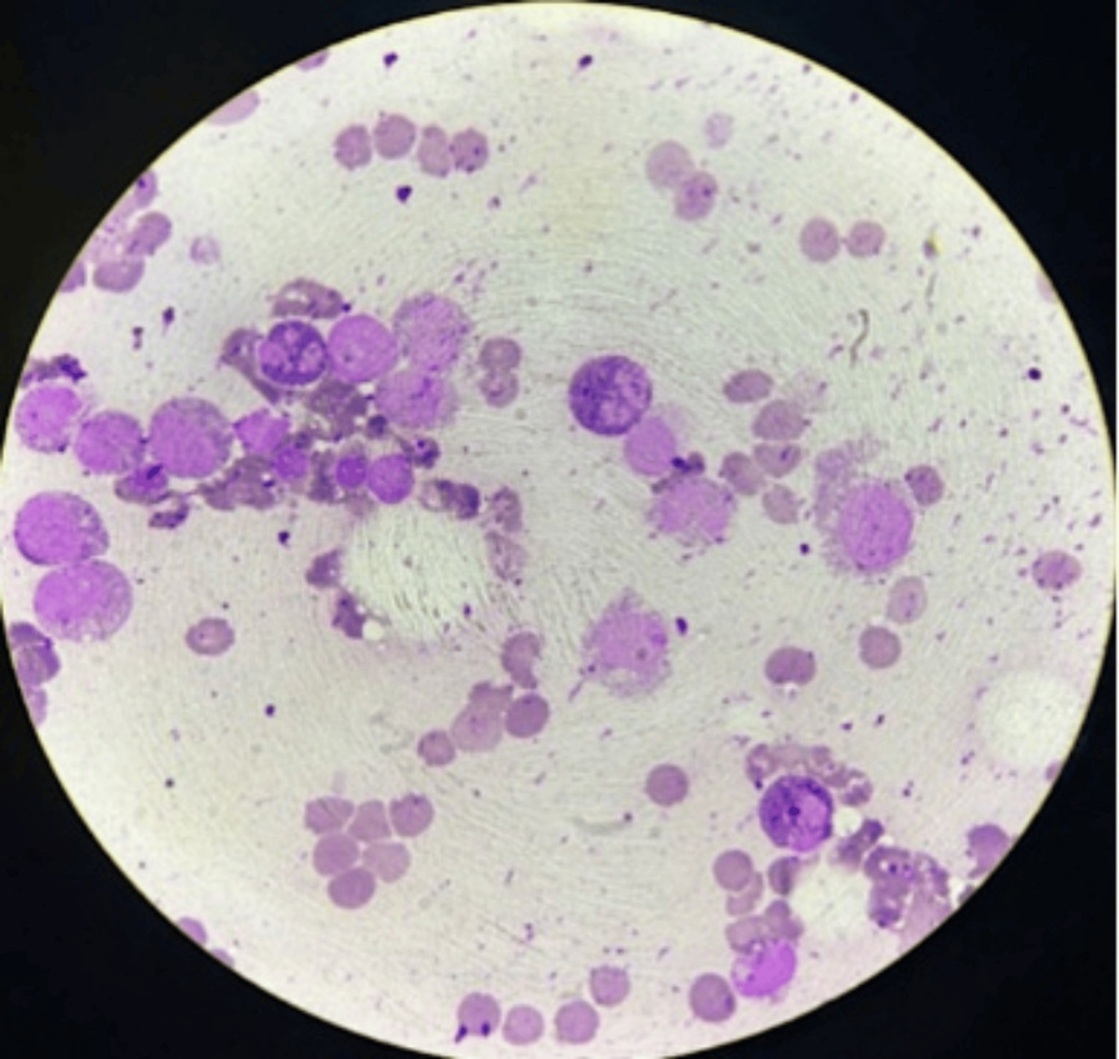
Bone marrow aspirate showing marked megakaryocytic hypoplasia.

We report the case of an 80-year-old woman with metabolic cirrhosis, portal hypertension, and hypersplenism, complicated by chronic thrombocytopenia and recurrent lower GI bleeding (LGIB) due to intestinal angiodysplasias. Her baseline platelet count was approximately 70 × 10⁹/L (reference range, 150-450 × 10⁹/L), occasionally decreasing to 40 × 10⁹/L. She was admitted with melena and severe thrombocytopenia of 10 × 10⁹/L. Platelet apheresis transiently increased the count to 26 × 10⁹/L, but transfusions were discontinued after two severe adverse reactions. The presence of wet purpura suggested an immune aetiology, prompting initiation of eltrombopag. Octreotide was introduced to control GI bleeding. Two days after starting octreotide, her platelet count fell to 14 × 10⁹/L, reaching a nadir of 1 × 10⁹/L by day nine. Bone marrow aspiration revealed a complete absence of megakaryocytes, raising suspicion for acquired amegakaryocytic thrombocytopenic purpura (AATP) (Figure 1). Bone marrow immunophenotyping showed that CD34+ cells demonstrated differentiation into various myeloid and lymphoid lineages, with normal maturation of the neutrophil, monocyte, and nucleated erythroid series. There was no phenotypic evidence of an aberrant population suggestive of blasts.

Cytogenetic analysis showed a normal female karyotype (46,XX). Laboratory tests demonstrated antiplatelet antibodies, along with decreased protein C and S levels (Table 1).

**Table 1:** Laboratory findings of the patient. IgG: Immunoglobulin G; IgM: Immunoglobulin M; IgA: Immunoglobulin A.

Laboratory test	Patient’s result	Reference range
Indirect IgG antiplatelet antibodies	Positive	Negative
Direct IgG antiplatelet antibodies	Positive	Negative
Indirect IgM antiplatelet antibodies	Negative	Negative
Indirect IgA antiplatelet antibodies	Positive	Negative
Protein C	63%	70-140%
Protein S	47%	63.5-149%
Karyotype	46,XX	46,XX = normal female karyotype; 46,XY = normal male karyotype

High-dose dexamethasone and intravenous immunoglobulin (IVIG) were administered, resulting in rapid improvement within 24 hours, likely reflecting both therapeutic effect and discontinuation of octreotide.

**Figure 2:**
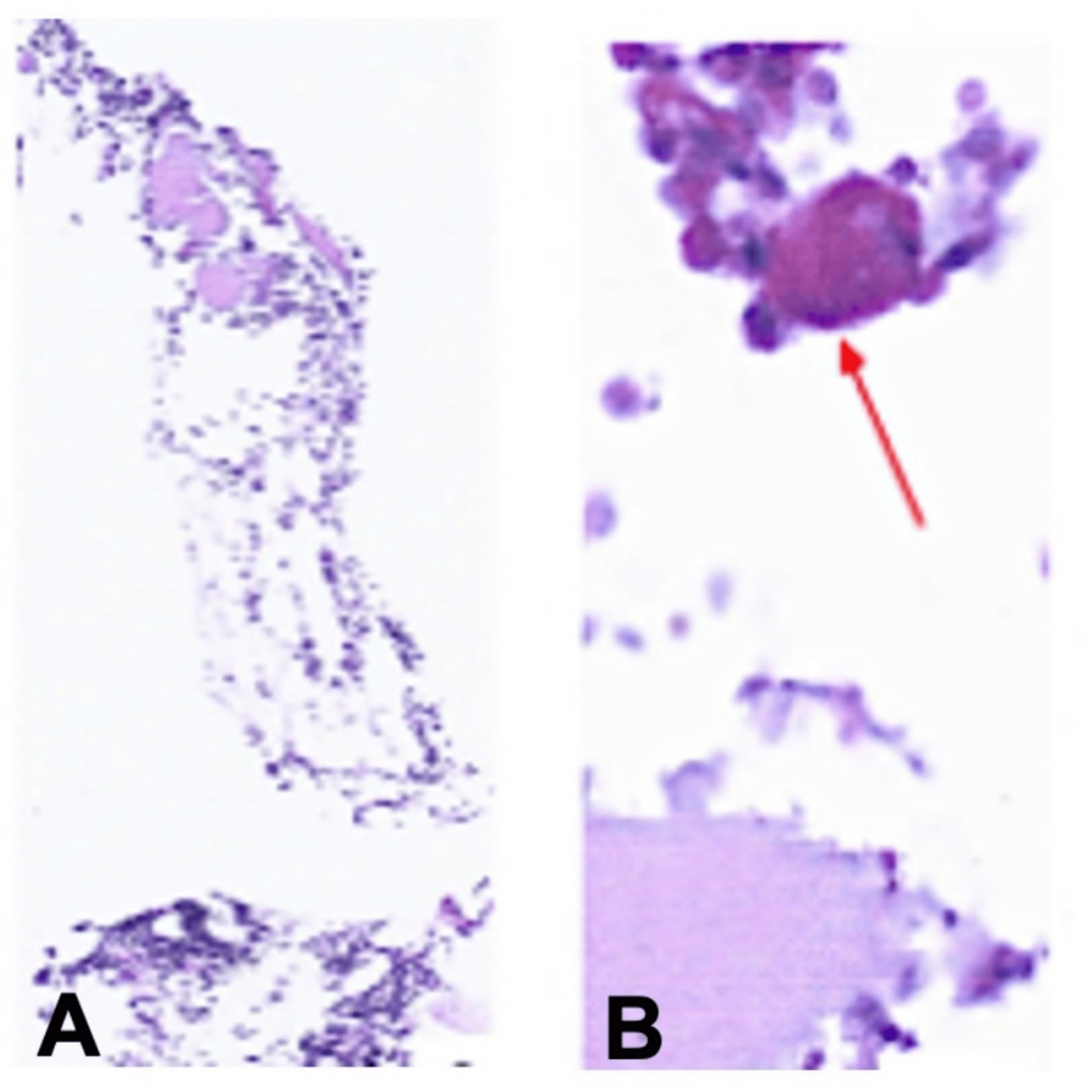
(A) Hematopoietic clusters exhibiting low cellularity. (B) A single megakaryocyte (indicated by the arrow) showing cytoplasmic periodic acid–Schiff (PAS) positivity without notable morphological abnormalities.

Her platelet count increased to 36 × 10⁹/L, accompanied by resolution of GI bleeding. She was discharged with a platelet count of 48 × 10⁹/L, and five days later her platelet count had risen to 183 × 10⁹/L. Eltrombopag was gradually tapered over 10 days due to the thrombotic risk associated with cirrhosis. The bone marrow biopsy sample was limited and insufficient to definitively confirm AATP (Figure 2).

**Figure 3:**
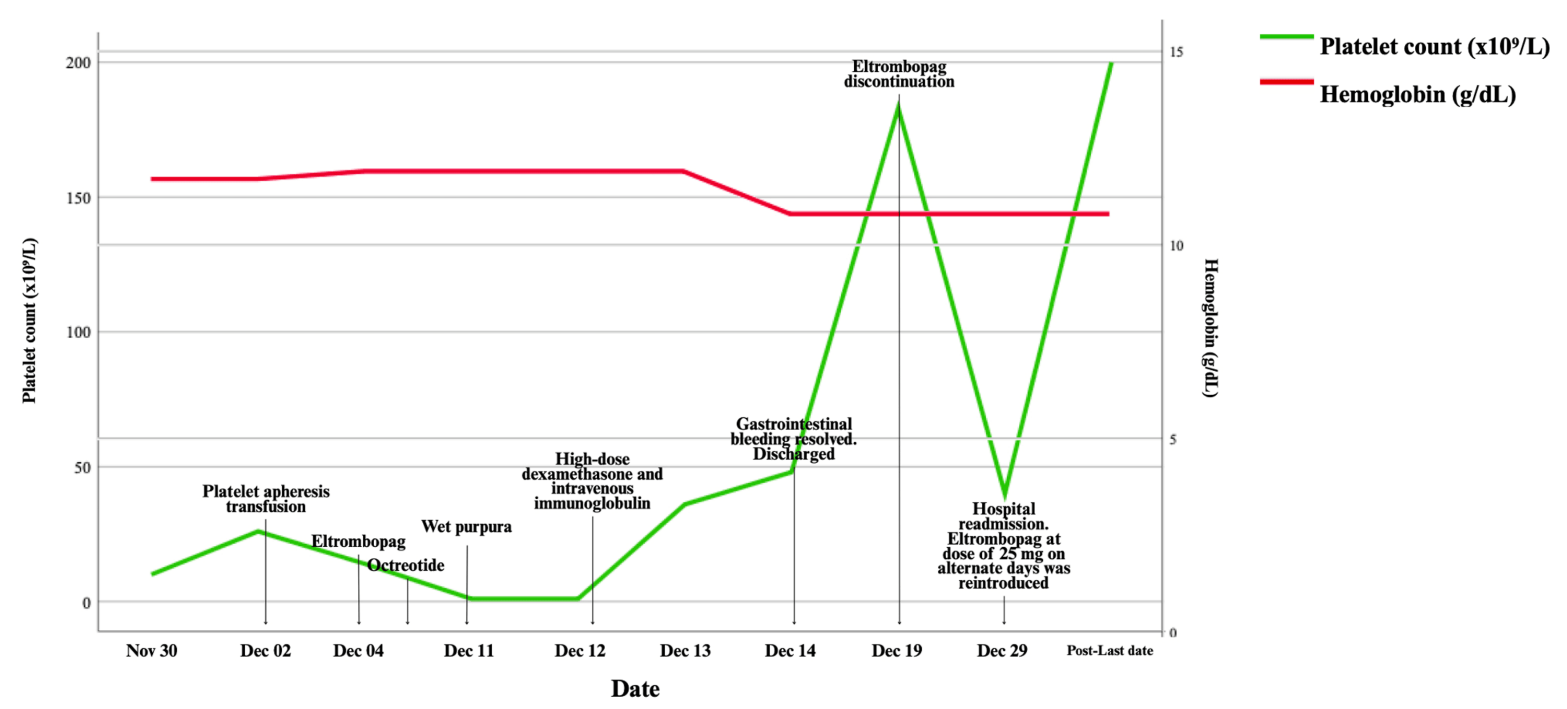
Platelet count and hemoglobin timeline.

However, the near-complete absence of megakaryocytes in the aspirate was notable and supported the initial findings, although this was not entirely consistent with a diagnosis of DIT. Ten days after discharge, the patient was readmitted with a platelet count of 40 × 10⁹/L due to recurrent LGIB. Eltrombopag at a dose of 25 mg on alternate days was reintroduced, and she achieved a sustained response with platelet counts reaching approximately 200 × 10⁹/L, a pattern more consistent with AATP or bone marrow hypoplasia than with DIT (Figure 3).

Since then, she has had additional episodes of LGIB from angiodysplasias requiring hospitalisation; however, her platelet counts remained stable around 200 × 10⁹/L during these episodes.

## Discussion

Thrombocytopenia in cirrhosis is frequently multifactorial, most often related to hypersplenism and diminished hepatic thrombopoietin synthesis [7]. However, the abrupt decline in platelet count following octreotide administration in our patient suggested a drug-induced mechanism [5,6]. DIT typically develops one to two weeks after exposure but may occur sooner in sensitized individuals [3]. In this setting, drug-dependent antibodies bind to platelet surface glycoproteins only in the presence of the sensitizing drug, leading to accelerated platelet destruction and, in some cases, impaired megakaryocyte maturation [2,3]. Although octreotide-induced DIT is exceedingly rare, a few cases have been documented [5,6]. The clinical presentation in our patient supported this diagnosis; however, bone marrow findings demonstrating marked megakaryocytic depletion were atypical, raising suspicion for AATP, a rare disorder characterized by isolated thrombocytopenia and absence of megakaryocytes in an otherwise normal marrow [7]. Although the sample was insufficient for definitive confirmation and showed hypocellularity, the rapid response to immunosuppressive therapy favoured an autoimmune process. AATP has diverse aetiologies, including autoimmune diseases, viral infections, and immunomodulatory drugs [8].

In our patient, thrombocytopenia recurred after eltrombopag discontinuation and improved upon reintroduction, consistent with bone marrow hypoplasia associated with cirrhosis and thrombopoietin deficiency. Bone marrow hypoplasia may initially mimic AATP and later evolve into overt marrow suppression. Cirrhotic patients exposed to myelotoxic drugs may experience impaired megakaryopoiesis, as illustrated in a similar case reported by Suyama T et al., where AATP developed after durvalumab therapy and responded to eltrombopag [9]. Likewise, eltrombopag was required in our patient to achieve sustained platelet recovery. Although this agent carries a thrombotic risk, particularly in cirrhotic patients with decreased protein C and S levels, the therapeutic benefit outweighed this concern. Its cautious use at adjusted doses has proven effective for optimizing platelet counts before invasive procedures [7]. Since thrombotic events have been reported when platelet counts exceed 200 × 10⁹/L, individualized dosing and close monitoring are essential to minimize risk.

## Conclusions

This case underscores the importance of maintaining clinical suspicion for immune-mediated thrombocytopenia in cirrhotic patients, even when bone marrow findings indicate hypoplasia or AATP. Drug-induced mechanisms may overlap with underlying marrow dysfunction, complicating both diagnosis and management. A comprehensive evaluation and timely initiation of immunosuppressive therapy, in addition to drug withdrawal, may be critical for achieving hematologic recovery and preventing severe bleeding complications.
